# The genome sequence of the Brindled Green, *Dryobotodes eremita* (Fabricius, 1775)

**DOI:** 10.12688/wellcomeopenres.19300.2

**Published:** 2026-02-18

**Authors:** Douglas Boyes, Peter W.H. Holland, University of Oxford and Wytham Woods Genome Acquisition Lab, Darwin Tree of Life Barcoding collective, Wellcome Sanger Institute Tree of Life programme, Wellcome Sanger Institute Scientific Operations: DNA Pipelines collective, Tree of Life Core Informatics collective, Darwin Tree of Life Consortium

**Affiliations:** 1 UK Centre for Ecology & Hydrology, Wallingford, England, UK; 2 University of Oxford, Oxford, England, UK

**Keywords:** Dryobotodes eremita, Brindled Green, genome sequence, chromosomal, Lepidoptera

## Abstract

We present a genome assembly from an individual female *Dryobotodes eremita* (the Brindled Green; Arthropoda; Insecta; Lepidoptera; Noctuidae). The genome sequence is 709.8 megabases in span. Most of the assembly is scaffolded into 32 chromosomal pseudomolecules including the Z and W sex chromosomes. The mitochondrial genome has also been assembled and is 15.5 kilobases in length. Gene annotation of this assembly on Ensembl identified 19,706 protein coding genes. This assembly was generated as part of the Darwin Tree of Life project, which produces reference genomes for eukaryotic species found in Britain and Ireland.

## Species taxonomy

Eukaryota; Metazoa; Ecdysozoa; Arthropoda; Hexapoda; Insecta; Pterygota; Neoptera; Endopterygota; Lepidoptera; Glossata; Ditrysia; Noctuoidea; Noctuidae; Xyleninae; *Dryobotodes*; *Dryobotodes eremita* (Fabricius, 1775) (NCBI:txid988106).

## Background

The Brindled Green, *Dryobotodes eremita*, is a small moth in the family Noctuidae (wingspan 32–39 mm) with a distinctive crinkled appearance to the forewings which are patterned with patches of green, black and cream overlain with rosy streaks. The variegated colouration extends to the thorax, head and legs such that the moth is cryptic when resting on lichen-covered tree trunks. The extent of green and red colouration is variable ( Bretherton *et al.*, 1983).

*D. eremita* is found across Europe and further east into Russia ( GBIF Secretariat, 2022). The species has an extensive distribution in Britain being found in woodland, parks and gardens across England, Wales, Scotland and Northern Ireland. Records range from Cornwall and the Scilly Isles in the south to Orkney and Shetland in the far north, although it is more common in the south of this range ( NBN Atlas Partnership, 2021). In Ireland the moth has been recorded across much of the country ( MothsIreland, 2022). The moth is on the wing in September and October in the south of England, or August and September in central and northern Scotland, and can be attracted to light or sugary substances ( Bretherton *et al.*, 1983; Randle *et al.*, 2019). Eggs are laid in autumn on pedunculate oak *Quercus robur* and when larvae hatch from eggs in spring they bore into an oak leaf-bud to feed. As the larvae develop, they switch to feeding on young leaves at terminal shoots ( Bretherton *et al.*, 1983; Stokoe, 1948). The species name *eremita*, meaning ‘hermit’, refers to the habit of the young larva living in a chamber formed by spinning oak leaves together with silk ( Emmet, 1991); later instars feed on oak leaves openly. Pupation occurs in soil near the base of oak trees ( Stokoe, 1948).

A genome sequence for *Dryobotodes eremita* will facilitate studies investigating molecular adaptations to oak feeding and will contribute to the growing set of genomic resources for Lepidoptera.

## Genome sequence report

**Figure 1.**
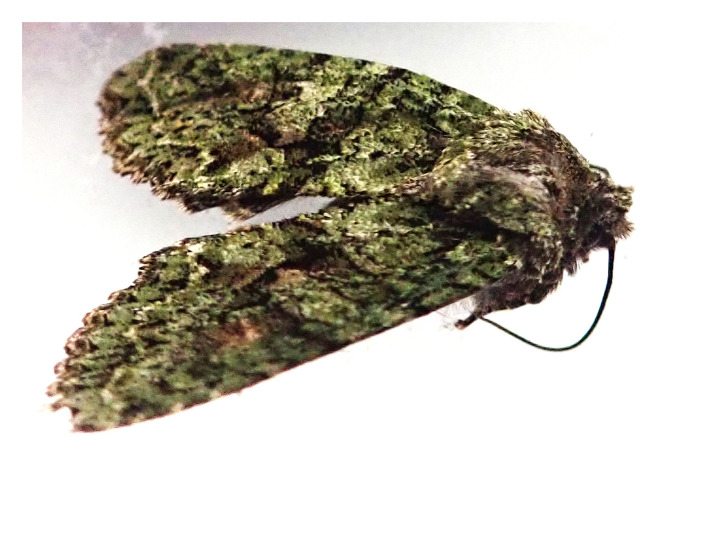
Photograph of the *Dryobotodes eremita* (ilDryErem1) specimen used for genome sequencing.

The genome was sequenced from one female *Dryobotodes eremita* ( Figure 1) collected from Wytham Woods, UK (latitude 51.77, longitude –1.34). A total of 28-fold coverage in Pacific Biosciences single-molecule HiFi long reads and 55-fold coverage in 10X Genomics read clouds were generated. Primary assembly contigs were scaffolded with chromosome conformation Hi-C data. Manual assembly curation corrected 40 missing joins or mis-joins and removed 10 haplotypic duplications, reducing the assembly length by 0.83% and the scaffold number by 33.66%, and increasing the scaffold N50 by 0.78%.

**Figure 2.**
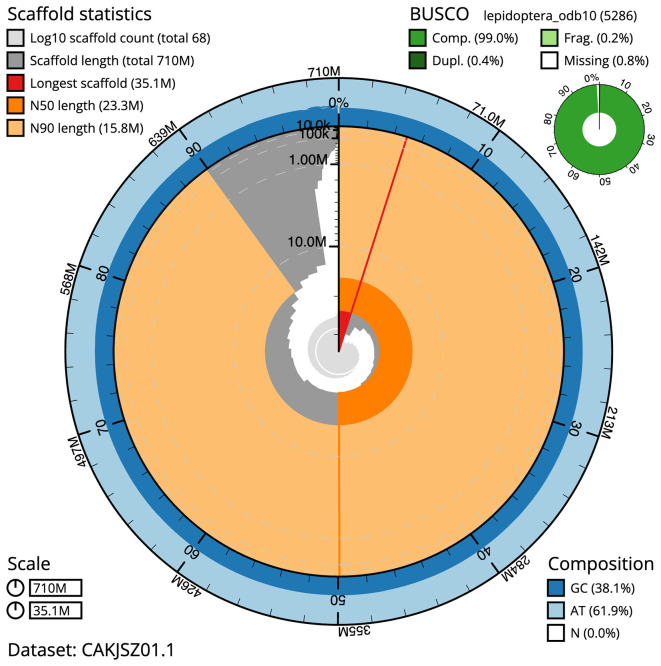
Genome assembly of *Dryobotodes eremita*, ilDryErem1.1: metrics. The BlobToolKit Snailplot shows N50 metrics and BUSCO gene completeness. The main plot is divided into 1,000 size-ordered bins around the circumference with each bin representing 0.1% of the 709,806,353 bp assembly. The distribution of scaffold lengths is shown in dark grey with the plot radius scaled to the longest scaffold present in the assembly (35,077,990 bp, shown in red). Orange and pale-orange arcs show the N50 and N90 scaffold lengths (23,327,181 and 15,843,934 bp), respectively. The pale grey spiral shows the cumulative scaffold count on a log scale with white scale lines showing successive orders of magnitude. The blue and pale-blue area around the outside of the plot shows the distribution of GC, AT and N percentages in the same bins as the inner plot. A summary of complete, fragmented, duplicated and missing BUSCO genes in the lepidoptera_odb10 set is shown in the top right. An interactive version of this figure is available at https://blobtoolkit.genomehubs.org/view/ilDryErem1.1/dataset/CAKJSZ01.1/snail.

The final assembly has a total length of 709.8 Mb in 67 sequence scaffolds with a scaffold N50 of 23.3 Mb ( Table 1). Most (99.8%) of the assembly sequence was assigned to 32 chromosomal-level scaffolds, representing 30 autosomes, and the W and Z sex chromosome. Chromosome-scale scaffolds confirmed by the Hi-C data are named in order of size ( Figure 2– Figure 5; Table 2). While not fully phased, the assembly deposited is of one haplotype. Contigs corresponding to the second haplotype have also been deposited.

**Table 1.** Genome data for *Dryobotodes eremita*, ilDryErem1.1.

**Project accession data**		
Assembly identifier	ilDryErem1.1	
Species	*Dryobotodes eremita*	
Specimen	ilDryErem1	
NCBI taxonomy ID	988106	
BioProject	PRJEB46319	
BioSample ID	SAMEA8603190	
Isolate information	ilDryErem1, female: thorax (genome sequencing), head (Hi-C scaffolding)	
Assembly metrics *		***Benchmark***
Consensus quality (QV)	Primary: 56.7; alternate: 57.0; combined: 56.9	≥ *40*
*k*-mer completeness	Primary: 71.73%; alternate: 67.65%; combined: 98.99%	≥ *95%*
BUSCO **	C:99.0%[S:98.7%,D:0.4%], F:0.2%,M:0.8%,n:5,286	*C* ≥ *95%*
Percentage of assembly mapped to chromosomes	99.8%	≥ *90%*
Sex chromosomes	Z and W chromosomes	*localised homologous pairs*
Organelles	Mitochondrial genome assembled	*complete single alleles*
**Raw data accessions**		
PacificBiosciences SEQUEL II	ERR6808003	
10X Genomics Illumina	ERR6688525–ERR6688528	
Hi-C Illumina	ERR6688524	
PolyA RNA-Seq Illumina	ERR9435006	
**Genome assembly**		
Assembly accession	GCA_917490735.1	
*Accession of alternate haplotype*	GCA_917490515.1	
Span (Mb)	709.8	
Number of contigs	104	
Contig N50 length (Mb)	22.6	
Number of scaffolds	67	
Scaffold N50 length (Mb)	23.3	
Longest scaffold (Mb)	35.1	
**Genome annotation**		
Number of protein-coding genes	19,706	
Number of transcripts	19,901	

* Assembly metric benchmarks are adapted from column VGP-2020 of “ Table 1: Proposed standards and metrics for defining genome assembly quality” from ( Rhie *et al.*, 2021).

** BUSCO scores based on the lepidoptera_odb10 BUSCO set using v5.3.2. C = complete [S = single copy, D = duplicated], F = fragmented, M = missing, n = number of orthologues in comparison. A full set of BUSCO scores is available at https://blobtoolkit.genomehubs.org/view/ilDryErem1.1/dataset/CAKJSZ01.1/busco.

**Figure 3.**
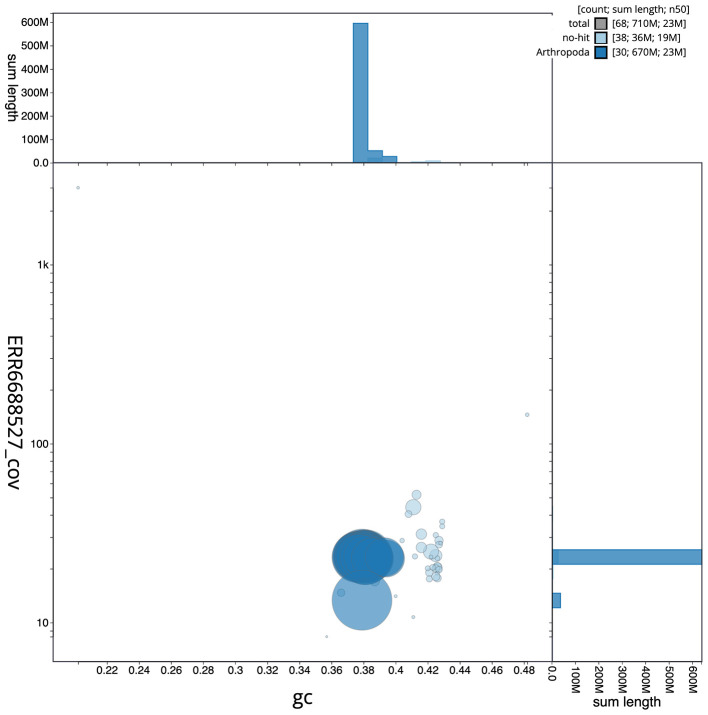
Genome assembly of *Dryobotodes eremita*, ilDryErem1.1: BlobToolKit GC-coverage plot. Scaffolds are coloured by phylum. Circles are sized in proportion to scaffold length. Histograms show the distribution of scaffold length sum along each axis. An interactive version of this figure is available at https://blobtoolkit.genomehubs.org/view/ilDryErem1.1/dataset/CAKJSZ01.1/blob.

**Figure 4.**
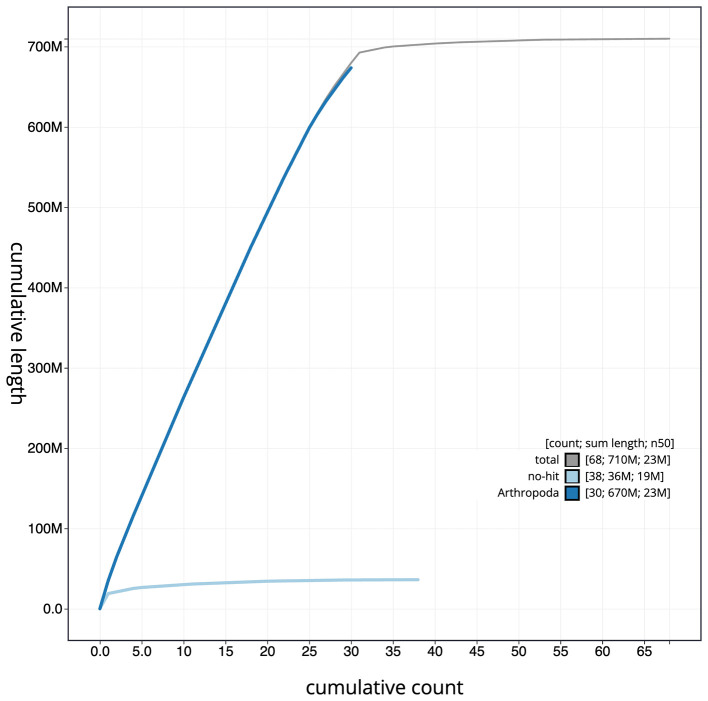
Genome assembly of *Dryobotodes eremita*, ilDryErem1.1: BlobToolKit cumulative sequence plot. The grey line shows cumulative length for all scaffolds. Coloured lines show cumulative lengths of scaffolds assigned to each phylum using the buscogenes taxrule. An interactive version of this figure is available at https://blobtoolkit.genomehubs.org/view/ilDryErem1.1/dataset/CAKJSZ01.1/cumulative.

**Table 2.** Chromosomal pseudomolecules in the genome assembly of *Dryobotodes eremita*, ilDryErem1.

INSDC accession	Chromosome	Size (Mb)	GC%
OU823242.1	1	29.33	38.1
OU823243.1	2	26.44	37.9
OU823244.1	3	25.13	37.7
OU823245.1	4	24.83	38.1
OU823246.1	5	24.77	38
OU823247.1	6	24.73	38.1
OU823248.1	7	24.58	37.6
OU823249.1	8	24.22	37.8
OU823250.1	9	23.99	38.1
OU823251.1	10	23.83	37.7
OU823252.1	11	23.47	37.7
OU823253.1	12	23.46	37.6
OU823254.1	13	23.33	38.1
OU823255.1	14	23.26	38
OU823256.1	15	23.21	38
OU823257.1	16	23.05	38
OU823258.1	17	22.99	37.7
OU823259.1	18	22.07	38.2
OU823260.1	19	22.01	38.1
OU823261.1	20	21.97	38
OU823262.1	21	21.65	38
OU823263.1	22	21.02	38.4
OU823264.1	23	20.32	38
OU823265.1	24	19.94	37.7
OU823266.1	25	18.97	38.3
OU823267.1	26	18.26	38.1
OU823268.1	27	17.13	38.6
OU823269.1	28	16.09	39.4
OU823270.1	29	15.84	38.5
OU823271.1	30	14.09	39.3
OU823272.1	W	9.31	38.1
OU823241.1	Z	35.08	37.9
OU823273.1	MT	0.02	20.3

**Figure 5.**
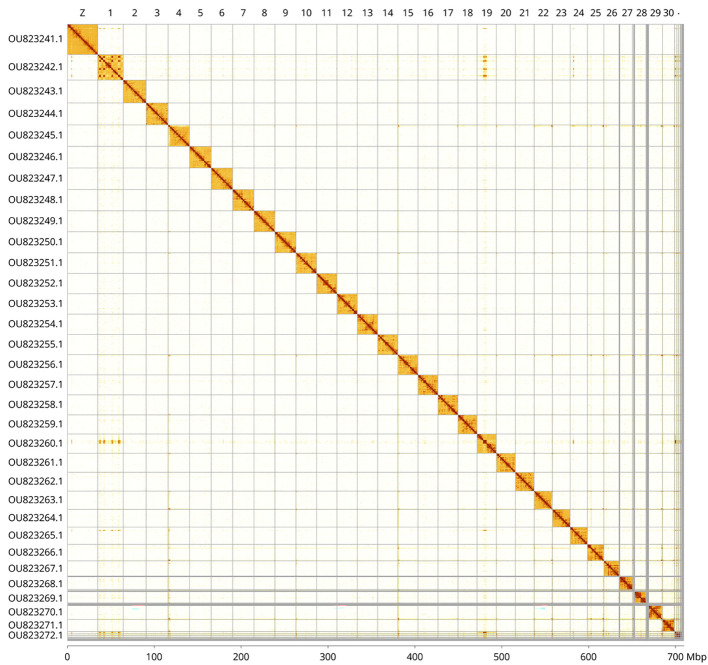
Hi-C contact map of the *Dryobotodes eremita* genome assembly. Assembled chromosomes are shown in order of size and labelled along the axes, with a megabase scale shown below. The plot was generated using PretextSnapshot. Chromosomes are shown in order of size from left to right and top to bottom. An interactive HiGlass version of this map may be viewed at https://genome-note-higlass.tol.sanger.ac.uk/l/?d=a-aARzNPStu0F6GjMD60XA.

The mitochondrial genome was also assembled (length 15.45 kb, OU823273.1). This sequence is included as a contig in the multifasta file of the genome submission and as a standalone record.

The combined primary and alternate assemblies achieve an estimated QV of 56.9. The *k*-mer completeness is 71.73% for the primary assembly, 67.65% for the alternate haplotype, and 98.99% for the combined assemblies. The primary assembly has a BUSCO v5.3.2 completeness of 99.0% (single = 98.7%, duplicated = 0.4%), using the lepidoptera_odb10 reference set ( *n* = 5,286).

## Genome annotation report

The *Dryobotodes eremita* genome assembly (GCA_917490735.1) was annotated by Ensembl at the European Bioinformatics Institute (EBI). This annotation includes 19,901 transcribed mRNAs from 19,706 protein-coding genes. The average transcript length is 7,516.09 bp, with an average of 5.15 exons per transcript. Further details of this annotation are available from the Ensembl annotation page.

## Methods

### Sample acquisition and nucleic acid extraction

A female *Dryobotodes eremita* (specimen number Ox000959, ToLID ilDryErem1) was collected from Wytham Woods, Oxfordshire (biological vice-county: Berkshire), UK (latitude 51.77, longitude –1.34) on 8 September 2020. The specimen was taken from woodland habitat by Douglas Boyes (University of Oxford) using a light trap. The specimen was identified by the collector and snap-frozen on dry ice.

Protocols for high molecular weight (HMW) DNA extraction developed at the Wellcome Sanger Institute (WSI) Tree of Life Core Laboratory are available on protocols.io ( Howard *et al.*, 2025).

DNA was extracted at the Tree of Life laboratory, Wellcome Sanger Institute (WSI). The ilDryErem1 sample was weighed and dissected on dry ice with head tissue set aside for Hi-C sequencing. Thorax tissue was cryogenically disrupted to a fine powder using a Covaris cryoPREP Automated Dry Pulveriser, receiving multiple impacts. High molecular weight (HMW) DNA was extracted using the Qiagen MagAttract HMW DNA extraction kit. Low molecular weight DNA was removed from a 20 ng aliquot of extracted DNA using the 0.8X AMpure XP purification kit prior to 10X Chromium sequencing; a minimum of 50 ng DNA was submitted for 10X sequencing. HMW DNA was sheared into an average fragment size of 12–20 kb in a Megaruptor 3 system with speed setting 30. Sheared DNA was purified by solid-phase reversible immobilisation using AMPure PB beads with a 1.8X ratio of beads to sample to remove the shorter fragments and concentrate the DNA sample. The concentration of the sheared and purified DNA was assessed using a Nanodrop spectrophotometer and Qubit Fluorometer and Qubit dsDNA High Sensitivity Assay kit. Fragment size distribution was evaluated by running the sample on the FemtoPulse system.

RNA was extracted from abdomen tissue of ilDryErem1 in the Tree of Life Laboratory at the WSI using TRIzol, according to the manufacturer’s instructions. RNA was then eluted in 50 μl RNAse-free water and its concentration assessed using a Nanodrop spectrophotometer and Qubit Fluorometer using the Qubit RNA Broad-Range (BR) Assay kit. Analysis of the integrity of the RNA was done using Agilent RNA 6000 Pico Kit and Eukaryotic Total RNA assay.

### Sequencing

Pacific Biosciences HiFi circular consensus and 10X Genomics read cloud DNA sequencing libraries were constructed according to the manufacturers’ instructions. Poly(A) RNA-Seq libraries were constructed using the NEB Ultra II RNA Library Prep kit. DNA and RNA sequencing was performed by the Scientific Operations core at the WSI on Pacific Biosciences SEQUEL II (HiFi), Illumina HiSeq 4000 (RNA-Seq) and Illumina NovaSeq 6000 (10X) instruments. Hi-C data were also generated from head tissue of ilDryErem1 using the Arima v2 kit and sequenced on the Illumina NovaSeq 6000 instrument.

### Genome assembly, curation and evaluation

Assembly was carried out with Hifiasm ( Cheng *et al.*, 2021) and haplotypic duplication was identified and removed with purge_dups ( Guan *et al.*, 2020). One round of polishing was performed by aligning 10X Genomics read data to the assembly with Long Ranger ALIGN, calling variants with FreeBayes ( Garrison & Marth, 2012). The assembly was then scaffolded with Hi-C data ( Rao *et al.*, 2014) using SALSA2 ( Ghurye *et al.*, 2019). The assembly was checked for contamination as described previously ( Howe *et al.*, 2021). Manual curation was performed using HiGlass ( Kerpedjiev *et al.*, 2018) and Pretext ( Harry, 2022). The mitochondrial genome was assembled using MitoHiFi ( Uliano-Silva *et al.*, 2022), which runs MitoFinder ( Allio *et al.*, 2020) and uses these annotations to select the final mitochondrial contig and to ensure the general quality of the sequence. To evaluate the assembly, Merqury.FK was used to estimate consensus quality (QV) scores and *k*-mer completeness ( Rhie *et al.*, 2020). The genome was analysed within the BlobToolKit environment ( Challis *et al.*, 2020) and BUSCO scores ( Manni *et al.*, 2021; Simão *et al.*, 2015) were calculated. Table 3 contains a list of software tool versions and sources.

**Table 3.** Software tools: versions and sources.

Software tool	Version	Source
BlobToolKit	4.0.7	https://github.com/blobtoolkit/blobtoolkit
BUSCO	5.3.2	https://gitlab.com/ezlab/busco
FreeBayes	1.3.1-17-gaa2ace8	https://github.com/freebayes/freebayes
Hifiasm	0.15.3-r339	https://github.com/chhylp123/hifiasm
HiGlass	1.11.6	https://github.com/higlass/higlass
Long Ranger ALIGN	2.2.2	https://support.10xgenomics.com/genome-exome/ software/pipelines/latest/advanced/other-pipelines
Merqury.FK	1.1.2	https://github.com/thegenemyers/MERQURY.FK
MitoHiFi	2	https://github.com/marcelauliano/MitoHiFi
PretextView	0.2	https://github.com/wtsi-hpag/PretextView
purge_dups	1.2.3	https://github.com/dfguan/purge_dups
SALSA	2.2	https://github.com/salsa-rs/salsa

### Ethics and compliance issues

The materials that have contributed to this genome note have been supplied by a Darwin Tree of Life Partner. The submission of materials by a Darwin Tree of Life Partner is subject to the Darwin Tree of Life Project Sampling Code of Practice. By agreeing with and signing up to the Sampling Code of Practice, the Darwin Tree of Life Partner agrees they will meet the legal and ethical requirements and standards set out within this document in respect of all samples acquired for, and supplied to, the Darwin Tree of Life Project. All efforts are undertaken to minimise the suffering of animals used for sequencing. Each transfer of samples is further undertaken according to a Research Collaboration Agreement or Material Transfer Agreement entered into by the Darwin Tree of Life Partner, Genome Research Limited (operating as the Wellcome Sanger Institute), and in some circumstances other Darwin Tree of Life collaborators.

## Data availability

European Nucleotide Archive: *Dryobotodes eremita* (brindled green). Accession number PRJEB46319; https://identifiers.org/ena.embl/PRJEB46319. The genome sequence is released openly for reuse. The *Dryobotodes eremita* genome sequencing initiative is part of the Darwin Tree of Life (DToL) project. All raw sequence data and the assembly have been deposited in INSDC databases. Raw data and assembly accession identifiers are reported in Table 1.

## Author information

Members of the University of Oxford and Wytham Woods Genome Acquisition Lab are listed here: https://doi.org/10.5281/zenodo.4789928.

Members of the Darwin Tree of Life Barcoding collective are listed here: https://doi.org/10.5281/zenodo.4893703.

Members of the Wellcome Sanger Institute Tree of Life programme are listed here: https://doi.org/10.5281/zenodo.4783585.

Members of Wellcome Sanger Institute Scientific Operations: DNA Pipelines collective are listed here: https://doi.org/10.5281/zenodo.4790455.

Members of the Tree of Life Core Informatics collective are listed here: https://doi.org/10.5281/zenodo.5013541.

Members of the Darwin Tree of Life Consortium are listed here: https://doi.org/10.5281/zenodo.4783558.
