# Supplementary material for: The genome sequence of the Brindled Green, Dryobotodes eremita (Fabricius, 1775)
Source: Wellcome Open Res. 2026 Feb 18;8:208. Originally published 2023 May 10. [Version 2] doi: 10.12688/wellcomeopenres.19300.2 (PMC12980082; doi:10.12688/wellcomeopenres.19300.2)
Supplement: Peer review history

# Reviewer report: Reviewer response for version 2

**DOI:** 10.21956/wellcomeopenres.28658.r148621

**Published:** 2026-03-14

**Authors:** Noémie M.-C. Hévin

**Affiliations:** 1 Université de Montpellier, Montpellier, France

The authors present a high-quality genome assembly for *Dryobotodes eremita*. This is a valuable contribution: the genome is robust, and the methodological pipeline is clearly described. I particularly appreciated the informative figures and the background section, which provides useful biological context on the species. I only have a few suggestions: 

1. A reference to this sentence could be added to the abstract: “A genome sequence for Dryobotodes eremita will facilitate studies investigating molecular adaptations to oak feeding and will contribute to the growing set of genomic resources for Lepidoptera”.
2. Keywords should be added
3. Authors should detail how they identified Z and W chromosomes
4. The manuscript would benefit substantially from a concise discussion section. This should include a brief comparison of the newly generated genome with what is already known for related genera regarding chromosome number, genome size, and genome composition.

Are sufficient details of methods and materials provided to allow replication by others?

Partly

Is the rationale for creating the dataset(s) clearly described?

Yes

Are the datasets clearly presented in a useable and accessible format?

Yes

Are the protocols appropriate and is the work technically sound?

Yes

Reviewer Expertise:

Genomics, macroevolution

I confirm that I have read this submission and believe that I have an appropriate level of expertise to confirm that it is of an acceptable scientific standard, however I have significant reservations, as outlined above.

# Reviewer report: Reviewer response for version 2

**DOI:** 10.21956/wellcomeopenres.28658.r148226

**Published:** 2026-03-11

**Authors:** Woo Jae Kim, Xinyue Zhou, Zekun Wu

**Affiliations:** 1 Center for Life Sciences, Harbin Institute of Technology Center for Life Sciences (Ringgold ID: 598430), Harbin, Heilongjiang, China; 2 Harbin Institute of Technology, Harbin, China

This article presents a high-quality, chromosome-scale genome assembly of the Brindled Green moth, Dryobotodes eremita. The authors adopted well-established, technically sound long-read sequencing and Hi-C scaffolding workflows, and generated a highly complete, robust genomic resource for this species. All relevant sequencing data and assembly results have been deposited in public databases for open access, the dataset is clearly structured, and the manuscript fits the publishing scope of the journal's Data Note format.I have no suggestions for revision or further improvement.

Are sufficient details of methods and materials provided to allow replication by others?

Partly

Is the rationale for creating the dataset(s) clearly described?

Yes

Are the datasets clearly presented in a useable and accessible format?

Yes

Are the protocols appropriate and is the work technically sound?

Yes

Reviewer Expertise:

Drosophila, Behavioral genetics, Neural circuits, Neuropeptide, Social behavior, Social neuroscience, fruit fly, Science and Society, Honey bee

We confirm that we have read this submission and believe that we have an appropriate level of expertise to confirm that it is of an acceptable scientific standard.

# Reviewer report: Reviewer response for version 2

**DOI:** 10.21956/wellcomeopenres.28658.r148615

**Published:** 2026-03-11

**Authors:** Melody Clark

**Affiliations:** 1 Natural Environment Research Council, Cambridge, UK

This data note describes the genome sequence of a female Brindled Green moth *Dryobotodes eremita*. The background biology and biogeography are well described, especially the early life stage reliance on oak trees. DNA extraction techniques, sequencing, assembly and annotation are well described and use referenced methodologies and are open source, as expected by the Darwin Tree of Life. The final assembly comprises 32 chromosomal pseudochromosomes including the Z and W, plus the mitochondrial genome. This genome which was constructed using a small level of manual annotation is of very high quality.

Are sufficient details of methods and materials provided to allow replication by others?

Yes

Is the rationale for creating the dataset(s) clearly described?

Yes

Are the datasets clearly presented in a useable and accessible format?

Yes

Are the protocols appropriate and is the work technically sound?

Yes

Reviewer Expertise:

transcriptomics and genomics of non-model species

I confirm that I have read this submission and believe that I have an appropriate level of expertise to confirm that it is of an acceptable scientific standard.

# Reviewer report: Reviewer response for version 2

**DOI:** 10.21956/wellcomeopenres.28658.r148618

**Published:** 2026-03-11

**Authors:** Wai Lok So

**Affiliations:** 1 The Chinese University of Hong Kong, Hong Kong, Hong Kong

The authors present a high-quality genome assembly of the Brindled Green, *Dryobotodes eremita*, as a part of Darwin Tree of Life project. The methods are standard, solid, clear and technically sound. The assembly was well assigned and annotated, including the Z and W sex chromosomes and a complete mitochondrial genome. The resultant resources are also transparent and accessible.

 The genome assembly is considered to be a very useful resource for research on Lepidoptera ecology and evolution, and other related research including lepidopteran molecular adaptations as the authors have suggested.

 I have no major concerns with the manuscript, except for the following two comments:

 1. Can a scale bar be provided in Figure 1?

 2. Can the authors state how the assembly of the sex chromosomes being validated? Are the sex chromosomes assembled by mapping to other reference genomes, or based on some marker genes with *de novo *assembly?

Are sufficient details of methods and materials provided to allow replication by others?

Yes

Is the rationale for creating the dataset(s) clearly described?

Yes

Are the datasets clearly presented in a useable and accessible format?

Yes

Are the protocols appropriate and is the work technically sound?

Yes

Reviewer Expertise:

evolution, genomics, arthropods

I confirm that I have read this submission and believe that I have an appropriate level of expertise to confirm that it is of an acceptable scientific standard.

# Reviewer report: Reviewer response for version 2

**DOI:** 10.21956/wellcomeopenres.28658.r148613

**Published:** 2026-03-09

**Authors:** Somjit Homchan

**Affiliations:** 1 Naresuan University, Tha Pho, Thailand

This article presents valuable data with high-quality genome assembly of an adult female moth, *Dryobotodes eremita*. The authors also detail a chromosome-scale genome assembly achieved using various tools. However, I have a couple of suggestions for improvement:

1. It would be helpful if the authors included a scale in the specimen images so the reader can imagine how big or small of the insect specimen.
2. Additionally, more detailed information about the RNA analysis would be beneficial. Rather than just outlining the RNA extraction methods, a clearer explanation of the process would help readers understand and replicate the RNA analysis. 
3. In figure 5 Hi-C contact map of the *Dryobotodes eremita* genome assembly, the chromosome W should be labeled next to the chromosome 30.

Are sufficient details of methods and materials provided to allow replication by others?

Partly

Is the rationale for creating the dataset(s) clearly described?

Yes

Are the datasets clearly presented in a useable and accessible format?

Yes

Are the protocols appropriate and is the work technically sound?

Yes

Reviewer Expertise:

Molecular Markers, Population Genetics, Cytogenetics

I confirm that I have read this submission and believe that I have an appropriate level of expertise to confirm that it is of an acceptable scientific standard, however I have significant reservations, as outlined above.

# Reviewer report: Reviewer response for version 1

**DOI:** 10.21956/wellcomeopenres.21383.r101162

**Published:** 2024-10-01

**Authors:** Woo Jae Kim, Xinyue Zhou, Zekun Wu

**Affiliations:** 1 Harbin Institute of Technology, Harbin, China; 2 Center for Life Sciences, Harbin Institute of Technology Center for Life Sciences (Ringgold ID: 598430), Harbin, Heilongjiang, China

The manuscript presents a high-quality de novo genome assembly of the adult female *Dryobotodes eremita* as part of the Darwin Tree of Life Project. Using good sequencing methods, including Pacific Biosciences HiFi long reads and Hi-C data, the authors assembled primary contigs into 32 chromosomal-level scaffolds, representing 30 autosomes and the W and Z sex chromosomes, with an N50 value of 23.3 Mb. The assembly achieves 99% completeness according to BUSCO, indicating a well-structured and robust genomic resource for this species. 

 However, there are a few areas that require further clarification:

 1. While the methods are generally well-articulated, several steps, particularly related to DNA extraction and tissue selection, could be elaborated on further. For instance, the manuscript mentions that thorax tissue was cryogenically disrupted for DNA extraction. It would be beneficial to clarify the rationale behind selecting thorax tissue and how this choice may have impacted the quality or integrity of the extracted DNA.

 2. The hermeneutics of the BlobToolKit snailplot remain obfuscated, rendering the comprehension of its semantic content arduous for the naıve observer. The intricacies of its representational schema are not readily accessible, thereby necessitating a specialized level of expertise for the accurate interpretation of its graphic expression.

 3. I note the presence of an unplaced scaffold measuring 14.73 Mbps in Table 2. Please elucidate the content of this scaffold, specifically whether it harbors any genes of functional importance. If so, please provide an explanation of their roles and potential significance. In the absence of such genes, please indicate whether further investigation or annotation of this scaffold is planned. Addressing this matter is crucial for assessing the comprehensiveness and applicability of the genome assembly.

 Additionally, I have a few broader suggestions and inquiries regarding the overall context of the study:

 1. I have observed that the Darwin Tree of Life Project has published several similar genome sequencing articles with consistent workflows. While I have no concerns about the quality of the genome assembly itself, as a reader, I would appreciate more context about the broader scope of the study.

 For example:

 How many insects have been sequenced, are plans in place to expand this dataset if necessary, and what role do Brindled Green play in the long-term insect genomics strategy of the Darwin Tree of Life Project?

Are sufficient details of methods and materials provided to allow replication by others?

Partly

Is the rationale for creating the dataset(s) clearly described?

Yes

Are the datasets clearly presented in a useable and accessible format?

Yes

Are the protocols appropriate and is the work technically sound?

Yes

Reviewer Expertise:

Drosophila, Behavioral genetics, Neural circuits, Neuropeptide, Social behavior, Social neuroscience, fruit fly, Science and Society, Honey bee

We confirm that we have read this submission and believe that we have an appropriate level of expertise to confirm that it is of an acceptable scientific standard, however we have significant reservations, as outlined above.

# Response

**Published:** 2026-02-05

**Authors:** Tree of Life Team Sanger

**Affiliations:** Wellcome Trust Sanger Institute, UK

We thank the reviewer for their comments and suggestions. We address each point below. **1. Tissue choice for DNA extraction** For Lepidoptera processed within the Darwin Tree of Life programme, a standardised tissue strategy is used where specimen size permits: thoracic tissue for high molecular weight DNA extraction, head tissue for Hi-C library preparation, and abdominal tissue for RNA sequencing. A reference to Howard *et al., *2025, in which the development of Tree of Life wet lab protocols is discussed has been added to the methods section. **2. Interpretation of the BlobToolKit snail plot** The BlobToolKit snail plot is a standard summary visualisation used across Darwin Tree of Life genome notes to present assembly size, contiguity, GC content, and BUSCO completeness as a high-level quality overview. **3. Unplaced sequence reported in Table 2** The value that was reported in Table 2 does not represent a single unplaced scaffold and has therefore been removed from the table. Rather, it combines the lengths of unlocalised scaffolds that could not be confidently ordered or oriented but are nonetheless assigned to chromosomes. For this assembly, 99.8% of the sequence is assigned to chromosomes. The revised Figure 5, generated using PretextSnapshot, shows these unlocalised scaffolds immediately after the chromosomes to which they belong. **Broader context of the study** This genome was generated as part of the Darwin Tree of Life Project, an ongoing initiative to produce high-quality reference genomes for all named eukaryotic species in Britain and Ireland using a consistent and scalable workflow. Genome notes in this series are intended to document individual assemblies rather than to provide a synthesis of project-wide progress or taxon-level strategy. We have added a short contextual sentence to the Background section. Reference: Howard C, Denton A, Jackson B, et al.: On the path to reference genomes for all biodiversity: Lessons learned and laboratory protocols created in the Sanger Tree of Life core laboratory over the first 2000 species. bioRxiv. 2025; https://doi.org/10.1101/2025.04.11.648334

# Reviewer report: Reviewer response for version 1

**DOI:** 10.21956/wellcomeopenres.21383.r94260

**Published:** 2024-08-22

**Authors:** Kay Lucek

**Affiliations:** 1 University of Neuchâtel, Neuchâtel, Switzerland

The authors present the chromosome level genome assembly of an apparently female specimen of the Brindled Green *Dryobotodes*. The assembly consists of 32 chromosomes. The assembly is highly complete as revealed by the high BUSCO score. Sequencing and genome assembly seem to have followed using established methods. The assembly has been generated using an established pipeline of the Darwin Tree of Life Consortium.

 Overall, this genome note leaves me with a lot of questions. For instance, RNA was extracted and sequenced, but no indication is given what it was used for. Similarly, 55-fold coverage in 10X Genomics read clouds were generated, but what for? The genome note lacks the basic information about the sex of the sequenced specimen. Agreeably the presence of a W chromosome suggests that it is a female, yet this could also simply stated. More importantly, Table 2 records an unplaced scaffold of 14.73 Mbps.

 No indication is given why this relatively large amount of data is unplaced.

 Nevertheless, the presented assembly will be of great value to study genome evolution in Lepidoptera.

Are sufficient details of methods and materials provided to allow replication by others?

No

Is the rationale for creating the dataset(s) clearly described?

Yes

Are the datasets clearly presented in a useable and accessible format?

Yes

Are the protocols appropriate and is the work technically sound?

Yes

Reviewer Expertise:

Speciation genomics

I confirm that I have read this submission and believe that I have an appropriate level of expertise to confirm that it is of an acceptable scientific standard, however I have significant reservations, as outlined above.
